# Supplementary figures and images for: Lung infection by Pseudomonas aeruginosa induces neuroinflammation and blood–brain barrier dysfunction in mice
Source: J Neuroinflammation. 2023 May 27;20:127. doi: 10.1186/s12974-023-02817-7 (PMC10223932; doi:10.1186/s12974-023-02817-7)

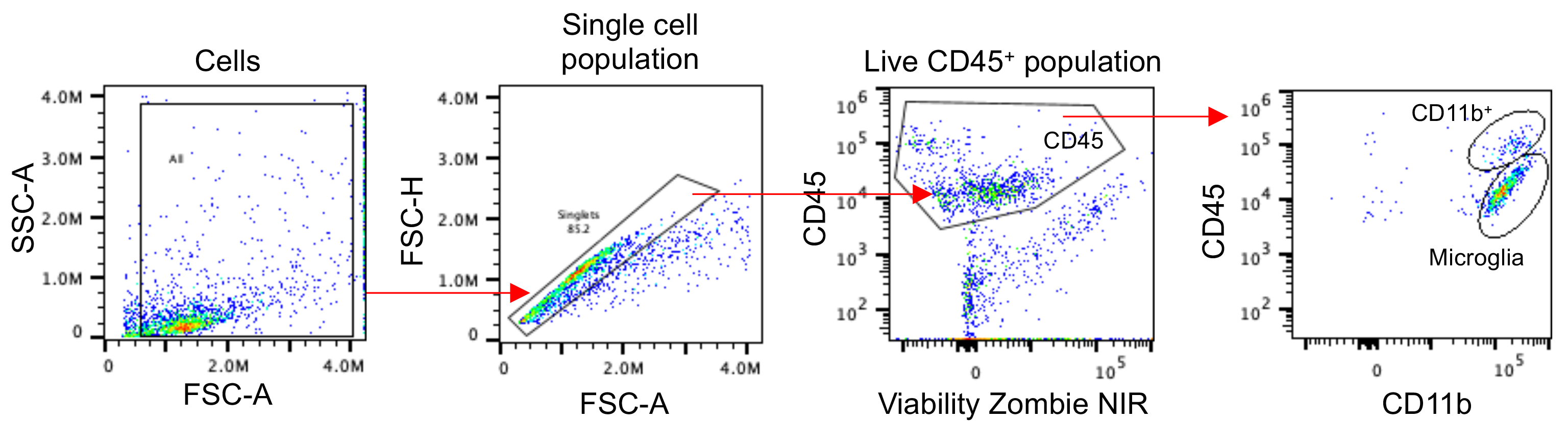

Supplement: Supplementary file 1 — Additional file 1: Figure S1. Representative flow cytometry plots illustrate the gating strategy used for this experimental series: single cell population was gated based on forward-scatter characteristics, Zombie viability dye was used to exclude dead cells, and CD45 and CD11b staining were used to identify myeloid leukocytesand microgliasubpopulation of cells. [file 12974_2023_2817_MOESM1_ESM.tif]

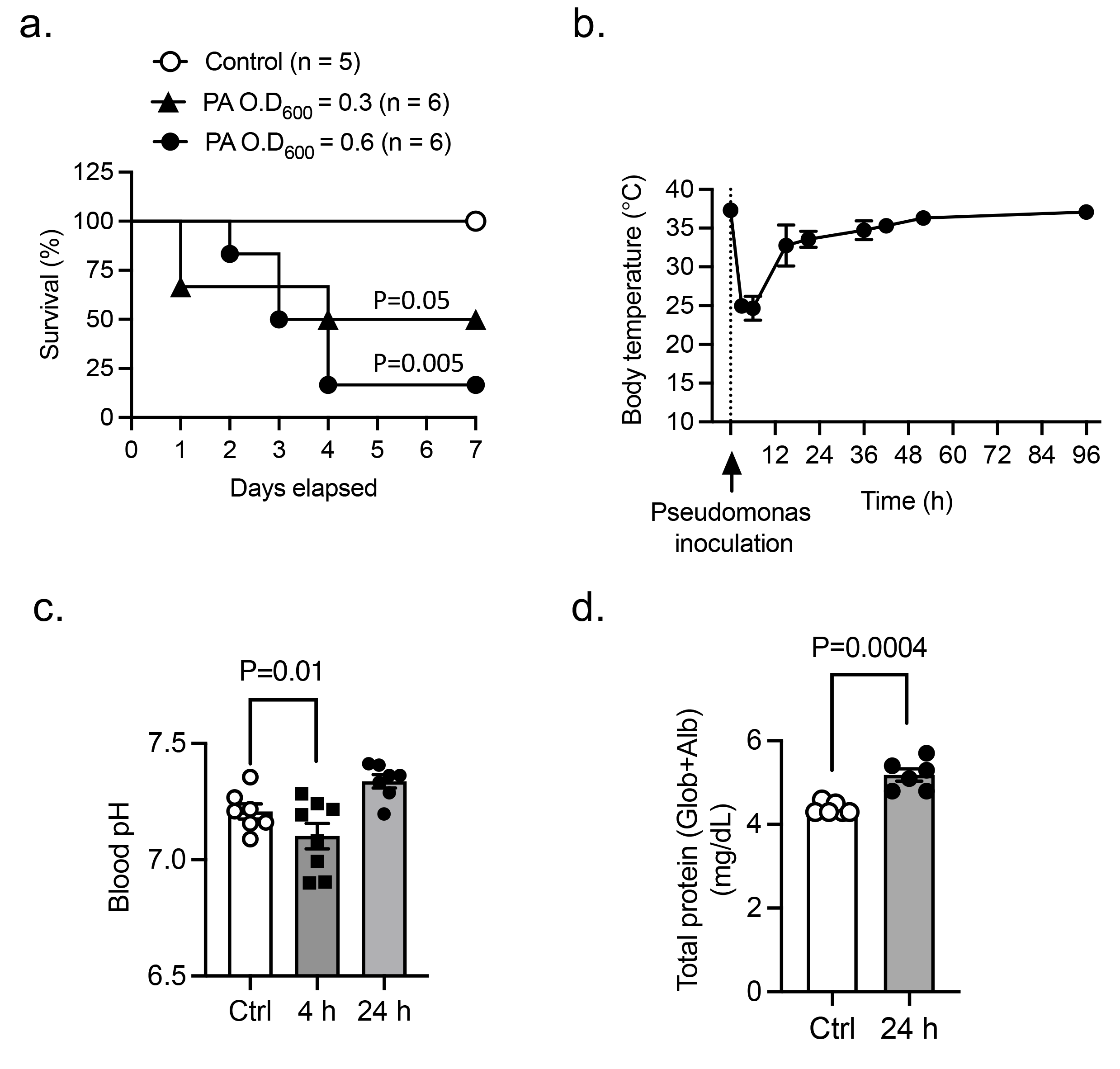

Supplement: Supplementary file 2 — Additional file 2: Figure S2. Effect of PA on mouse survival, body temperature and biochemistry parameters. Survival rates of mice treated with PA at OD600 0.3 and 0.6. Body temperature obtained by measuring the rectal temperature after PA infection. Blood pH values obtained from control and PA-infected mice at 4 h and 24 h after infection measured with iSTAT. Total proteinconcentration in blood measured in controls and 24-h after PA infection. [file 12974_2023_2817_MOESM2_ESM.tif]

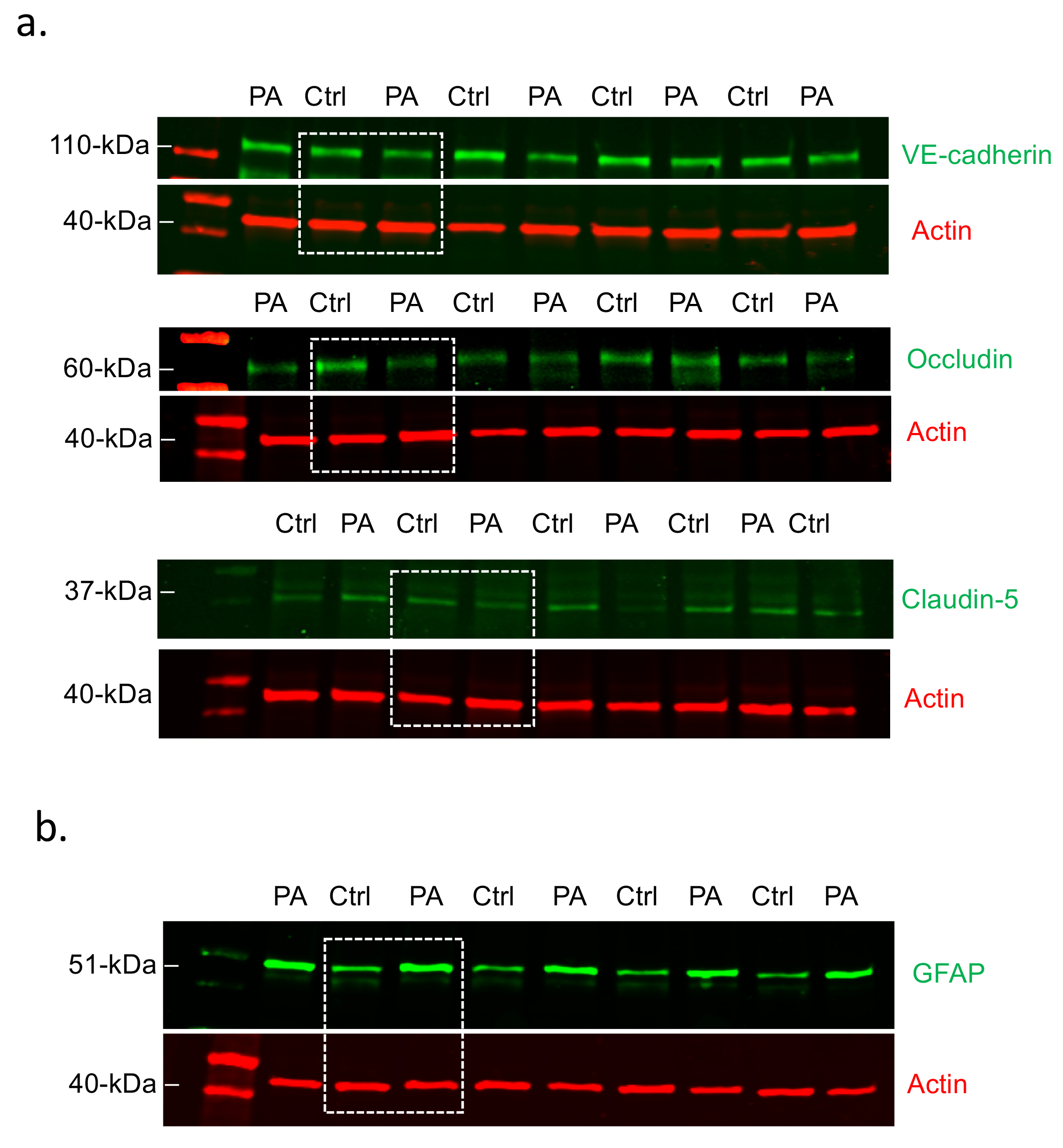

Supplement: Supplementary file 3 — Additional file 3: Figure S3. Full scans of all Western blots used for quantification in main figures.Western blots of VE-cadherin, occludin and claudin-5 in cerebral cortex homogenates from controlsand infected mice at 24 h post-infection. Western blots of GFAP in cerebral cortex homogenates from controlsand infected mice at 24 h post-infection. Dotted boxes indicate lanes presented as representative blots in the respective main figures. [file 12974_2023_2817_MOESM3_ESM.tif]
